# A Bacteriophage T4 Nanoparticle-Based Dual Vaccine against Anthrax and Plague

**DOI:** 10.1128/mBio.01926-18

**Published:** 2018-10-16

**Authors:** Pan Tao, Marthandan Mahalingam, Jingen Zhu, Mahtab Moayeri, Jian Sha, William S. Lawrence, Stephen H. Leppla, Ashok K. Chopra, Venigalla B. Rao

**Affiliations:** a Department of Biology, The Catholic University of America, Washington, DC, USA; b Microbial Pathogenesis Section, Laboratory of Parasitic Diseases, NIAID, NIH, Bethesda, Maryland, USA; c Department of Microbiology and Immunology, University of Texas Medical Branch, Galveston, Texas, USA; d Galveston National Laboratory, University of Texas Medical Branch, Galveston, Texas, USA; e Institute for Human Infections and Immunity, University of Texas Medical Branch, Galveston, Texas, USA; f Sealy Institute for Vaccine Sciences, University of Texas Medical Branch, Galveston, Texas, USA; g Center for Biodefense and Emerging Infectious Diseases, University of Texas Medical Branch, Galveston, Texas, USA; McGovern Medical School; University of Colorado Denver; University of Missouri; University of North Dakota

**Keywords:** anthrax vaccine, bacteriophage T4, biodefense, plague vaccine, small outer capsid protein, vaccine delivery, virus nanoparticles

## Abstract

Bacillus anthracis and Yersinia pestis, the causative agents of anthrax and plague, respectively, are two of the deadliest pathogenic bacteria that have been used as biological warfare agents. Although Biothrax is a licensed vaccine against anthrax, no Food and Drug Administration-approved vaccine exists for plague. Here, we report the development of a dual anthrax-plague nanoparticle vaccine employing bacteriophage (phage) T4 as a platform. Using an *in vitro* assembly system, the 120- by 86-nm heads (capsids) of phage T4 were arrayed with anthrax and plague antigens fused to the small outer capsid protein Soc (9 kDa). The antigens included the anthrax protective antigen (PA) (83 kDa) and the mutated (mut) capsular antigen F1 and the low-calcium-response V antigen of the type 3 secretion system from Y. pestis (F1mutV) (56 kDa). These viral nanoparticles elicited robust anthrax- and plague-specific immune responses and provided complete protection against inhalational anthrax and/or pneumonic plague in three animal challenge models, namely, mice, rats, and rabbits. Protection was demonstrated even when the animals were simultaneously challenged with lethal doses of both anthrax lethal toxin and Y. pestis CO92 bacteria. Unlike the traditional subunit vaccines, the phage T4 vaccine uses a highly stable nanoparticle scaffold, provides multivalency, requires no adjuvant, and elicits broad T-helper 1 and 2 immune responses that are essential for complete clearance of bacteria during infection. Therefore, phage T4 is a unique nanoparticle platform to formulate multivalent vaccines against high-risk pathogens for national preparedness against potential bioterror attacks and emerging infections.

## Abstract (precis)

Following the deadly anthrax attacks of 2001, the Centers for Disease Control and Prevention (CDC) determined that Bacillus anthracis and Yersinia pestis that cause anthrax and plague, respectively, are two Tier 1 select agents that pose the greatest threat to the national security of the United States. Both cause rapid death, in 3 to 6 days, of exposed individuals. We engineered a virus nanoparticle vaccine using bacteriophage T4 by incorporating key antigens of both B. anthracis and Y. pestis into one formulation. Two doses of this vaccine provided complete protection against both inhalational anthrax and pneumonic plague in animal models. This dual anthrax-plague vaccine is a strong candidate for stockpiling against a potential bioterror attack involving either one or both of these biothreat agents. Further, our results establish the T4 nanoparticle as a novel platform to develop multivalent vaccines against pathogens of high public health significance.

## INTRODUCTION

Vaccines are one of the most successful medical interventions of the past millennium (1). Millions of lives have been saved by mass administration of vaccines against deadly pathogens such as smallpox and flu. However, effective vaccines are still lacking for many pathogens, including biothreat agents such as the Gram-positive bacterium Bacillus anthracis that causes anthrax and the Gram-negative bacterium Yersinia pestis that causes plague. According to the Centers for Disease Control and Prevention (CDC), these two organisms are two of the Tier 1 select agents that pose the greatest threat to national security (2). Both are highly virulent resulting in mortality (as high as 100%) of the subjects within 3 to 6 days of infection. The organisms can be weaponized and transmitted through inhalation of aerosolized droplets and can be disseminated relatively easily for the purposes of biological warfare or bioterrorism (3, 4). Designing a vaccine that can protect the public against these threats is therefore a national priority. Additionally, plague is a significant threat to global health. It causes periodic outbreaks around the world. The latest example is the 2017 Madagascar outbreak that resulted in 209 deaths (>70% of the cases were pneumonic plague) (5).

Vaccine development historically relied on the whole pathogen containing either the inactivated (heat- or formalin-killed) organisms or live attenuated (less virulent mutant) organisms (1). Several such vaccines have been developed against anthrax and plague in the past. The Biothrax anthrax vaccine (AVA [anthrax vaccine alum-adsorbed]) is derived from the culture filtrate of the attenuated B. anthracis strain V770-NP1-R (6, 7). This strain secretes the anthrax protective antigen (PA), a primary target for anthrax vaccine design, into the culture medium, but also traces of the other two components of the tripartite anthrax toxin, the lethal factor (LF) and the edema factor (EF) (6–8). This vaccine does have untoward side effects, and therefore, new anthrax vaccines are highly sought. Similarly, plague vaccines were developed using the formalin-killed or live attenuated Yersinia pestis bacteria (9–11). However, these whole-pathogen vaccines produce severe side effects such as reactogenicity at the injection site. Hence, these vaccines have either been discontinued or used only in a limited way to protect at-risk military and laboratory personnel (6, 10–12).

Subunit vaccines containing only the target antigen(s) of a pathogen are safer alternatives to whole-pathogen vaccines (13, 14). Numerous recombinant vaccines against anthrax or plague have been under investigation, but none have yet been licensed (6, 15–18). The recombinant anthrax vaccines are composed primarily of PA (6, 18), whereas the plague vaccines contain two surface antigens of Y. pestis, the capsular protein Caf1 (or F1) (15.6 kDa) and the low-calcium-response V antigen LcrV (or V) (37.2 kDa) (15–17). A bivalent vaccine consisting of all three antigens fused into a single polyprotein has also been developed (19). However, the subunit vaccines generally are unstable, may not generate sufficient immune responses for complete protection, and require the addition of nonspecific “adjuvants” such as aluminum hydroxide or liposomes to enhance immunogenicity and protective efficacy (13, 20–22).

One of the reasons for poor immunogenicity of subunit vaccines is the lack of the pathogen-associated molecular patterns (PAMPs) observed in whole-pathogen vaccines, which are recognized by the host immune system and trigger innate as well as adaptive immune responses (22, 23). One way to overcome this limitation is to incorporate the target antigen as part of a virus nanoparticle structure (24–27). Phages, such as T4, lambda, and M13, have been used as viral nanoparticle platforms to display antigens (28–30). The antigen molecules so arrayed on the nanoparticle surface would mimic the PAMPs of a pathogenic virus, potentially leading to stimulation of strong immune responses (24, 25).

**FIG 1:**
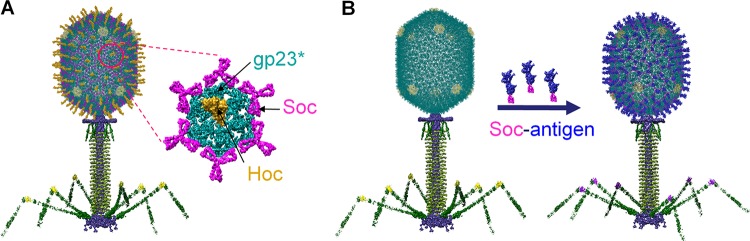
Schematic of the bacteriophage T4 nanoparticle platform. (A) Structural model of phage T4. The enlarged capsomer shows the major capsid protein gp23* (cyan) (the asterisk shows that it is the cleaved form) (930 copies), Soc (magenta) (870 copies), and Hoc (yellow) (155 copies). (B) *In vitro* assembly of Soc-fused antigen (blue) molecules on *hoc*^−^ *soc*^−^ T4 phage capsid.

Here, we report the development of a dual viral nanoparticle vaccine against both anthrax and plague using bacteriophage (phage) T4 (Fig. 1). The 120- by 86-nm-size phage T4 head (capsid) is decorated with anthrax PA (83 kDa) and plague F1mutV (56 kDa) fused to the small outer capsid protein (Soc) (9 kDa). F1mutV is a fusion protein of a mutant F1 antigen and V antigen. The mutant F1 produces a soluble monomeric protein, as opposed to the native F1 which polymerizes into heterogeneous aggregates, yet it retains the full immunogenicity of the native protein (17). Since Soc assembles on phage T4 capsid as a trimer at the quasi-three-fold axes (Fig. 1A) (31, 32), the PA and F1mutV antigens attached to Soc might be recognized by the host immune system as repeating structures arranged symmetrically on the nanoparticle (Fig. 1B), similar to PAMPs. Indeed, our studies demonstrated that such nanoparticles generated robust antigen-specific immune responses and provided complete protection against both anthrax and plague in three different animal models, namely, mice, rats, and rabbits. Furthermore, the T4 nanoparticle vaccine, unlike the traditional subunit vaccines, do not require any adjuvant and generated balanced T_H_1- and T_H_2-based antibody responses, which are highly desirable for any vaccine but particularly relevant for clearance of pathogenic plague bacteria (10, 33). Finally, the T4 nanoparticle vaccine provided complete protection against simultaneous challenge by both anthrax lethal toxin (LeTx) and Y. pestis CO92. These results suggest that the phage T4 vaccine might be a good candidate for stockpiling against a potential bioterror attack involving either one or both of these biothreat agents. Further, our results establish the T4 nanoparticle as a novel platform to develop multivalent biodefense vaccines containing additional biothreat antigens, as well as for engineering vaccines against other emerging pathogens of high public health significance.

## RESULTS

### Preparation of T4 nanoparticles decorated with anthrax and plague antigens.

To develop a phage T4 vaccine against both B. anthracis and Y. pestis, we constructed three recombinants by fusing the anthrax and plague antigens to phage RB69 Soc. The three recombinants were F1mutV-Soc-PA (148 kDa), F1mutV-Soc (66 kDa), and Soc-PA (93 kDa). Our previous studies have shown that both the N and C termini of RB69 Soc are exposed on the capsid surface, and both can be used to display recombinant proteins efficiently (34, 35). Phage RB69 is closely related to T4, and its Soc protein binds to phage T4 capsid as well as the T4 Soc protein does (34). As reported previously (34), the binding affinity, copy number per capsid, and capsid stabilization by Soc binding are nearly the same for RB69 Soc as those of T4 Soc. However, we observed that proteins fused to RB69 Soc showed greater solubility than the proteins fused to T4 Soc (17). Since such solubility is a significant factor in vaccine manufacture, we used RB69 Soc instead of T4 Soc for antigen display. The choice of the plague antigen F1mutV and the anthrax antigen PA was based on our previous studies in which these antigens stimulated protective immune responses in animal models (17, 19, 36–38). The His-tagged recombinant proteins were overexpressed in Escherichia coli and purified by immobilized nickel affinity chromatography followed by size exclusion chromatography (see Fig. S1 in the supplemental material). The purified proteins were then assembled on T4 nanoparticles in three different display formats: (i) display of F1mutV-Soc-PA, (ii) display of F1mutV-Soc and Soc-PA on the same capsid, and (iii) a 1:1 mixture of T4 phage particles separately displayed with F1mutV-Soc or Soc-PA. Of these display formats, the latter produced particles with the highest copy number of antigens per capsid, whereas F1mutV-Soc-PA produced the lowest copy number. Hence, this formulation, a mixture of T4 nanoparticles displaying either F1mutV-Soc or Soc-PA (abbreviated as T4-F1mutV/PA) was selected for immunological studies.

10.1128/mBio.01926-18.1 FIG S1 Purification of recombinant anthrax and plague antigens. Size exclusion chromatography profiles of F1mutV-Soc (A), Soc-PA (B), and F1mutV-Soc-PA (C) proteins. The recombinant proteins were purified from the cell-free lysates by HisTrap affinity chromatography followed by HiLoad 16/60 Superdex 200 size exclusion chromatography. The insets show the SDS-polyacrylamide gels of purified proteins. Download FIG S1, TIF file, 2 MB. Copyright © 2018 Tao et al. 2018 Tao et al. This content is distributed under the terms of the Creative Commons Attribution 4.0 International license.

**FIG 2:**
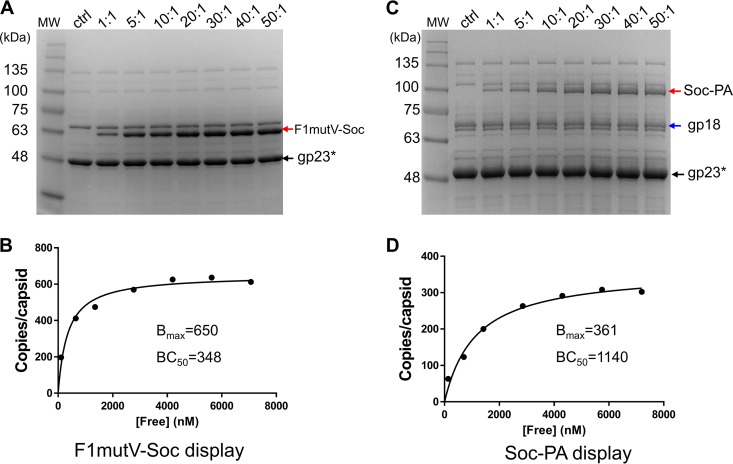
Display of F1mutV-Soc (A and B) and Soc-PA (C and D) on Hoc^−^ Soc^−^ phage T4. Approximately 2 × 10^10^ purified Hoc^−^ Soc^−^ phage particles were incubated at the indicated ratios of F1mutV-Soc or Soc-PA molecules to Soc binding sites in 200 µl PBS buffer (pH 7.4). The control (ctrl) lane contains the Hoc^−^ Soc^−^ phage. F1mutV-Soc (A) and Soc-PA (C) bands, which are not present in the phage control, are labeled with red arrows. (B and D) Saturation binding curves of F1mutV-Soc (B) and Soc-PA (D). The concentrations (in nanomolar) of free protein (F1mutV-Soc and Soc-PA) are shown on the *x* axes. The copy numbers of the capsid-bound antigens were determined using the major capsid protein gp23* (molecular weight [MW], 49 kDa; 930 copies per capsid) (black arrow) and the tail sheath protein gp18 (MW, 72 kDa; 138 copies per phage) as internal controls (blue arrows). The data were plotted as one-site saturation ligand binding curve, and the calculated binding parameters are shown. BC_50_, half-maximal binding concentration (in nanomolar); *B*_max_, maximum copy number per phage particle (see Materials and Methods for details).

To optimize the copy number for immunization experiments, 2 × 10^10^ particles of purified Soc^−^ (and Hoc^−^) phage were incubated with F1mutV-Soc or Soc-PA proteins at different ratios of antigen molecules to Soc binding sites (Fig. 1B and 2). Binding increased with increasing ratio, reaching saturation at ∼20:1 (Fig. 2). The copy numbers of antigens displayed per capsid (*B*_max_) were 650 for the 66-kDa F1mutV-Soc and 361 for the 93-kDa Soc-PA, and the binding concentrations at which half of the capsid binding sites were occupied (BC_50_s) were 348 nM and 1,140 nM, respectively (Fig. 2; see Materials and Methods for details on the determination of *B*_max_ and BC_50_). Since there are 870 Soc binding sites per capsid, the percent occupancy values were 75 for F1mutV-Soc and 41 for Soc-PA. These values represent high occupancies, considering that both the 83-kDa PA and the 56-kDa F1mutV would encounter steric constraints to access all the Soc binding sites on the capsid surface, the former more so than the latter, as reflected in the data.

### The T4 nanoparticles decorated with anthrax and plague antigens provide near-complete protection to mice against anthrax lethal toxin and pneumonic plague challenges with Y. pestis CO92.

**FIG 3:**
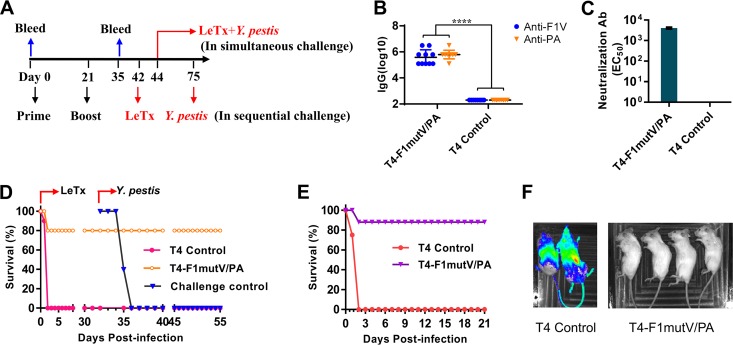
(A) Immunization scheme for mouse study. Mice were immunized (i.m.) on days 0 and 21. Sera were collected on days 0 and 35 for antibody analysis. In a sequential challenge model, animals were challenged with LeTx on day 42 followed by Y. pestis CO92 on day 75. In a simultaneous challenge model, mice were challenged with LeTx and Y. pestis CO92 on day 44. (B) Antigen-specific total IgG titers. Each symbol represents the value for an individual mouse. Values that are significantly different (*P* < 0.0001 by Student’s *t* test) are indicated by a bar labeled with four asterisks. (C) LeTx neutralizing antibody titers. Error bars represent standard deviations. Ab, antibodies; EC_50_, effective serum concentration inducing 50% neutralization. (D) Survival of mice against anthrax LeTx and plague sequential challenge. (E) Survival of mice against simultaneous anthrax LeTx and plague challenge. (F) *In vivo* imaging of mice challenged with Y. pestis CO92 expressing luciferase. The animal survival data are representative of two biological replicates. Representative mice from control group (two mice) and T4-F1mutV/PA-immunized group (four mice) were shown.

BALB/c mice (10 mice per group) were immunized by the intramuscular (i.m.) route with 25 µg of each of the T4 nanoparticle preparations containing F1mutV-Soc and Soc-PA and boosted on day 21 (Fig. 3A). Mice immunized with the T4 phage lacking the antigens served as a control group. A series of experiments were performed to determine the immunogenicity and protective efficacy of the T4-delivered antigens. Enzyme-linked immunosorbent assays (ELISAs) showed high levels of both F1V-specific and PA-specific IgG antibodies, up to endpoint titers of ∼3 × 10^6^ (Fig. 3B). Anthrax lethal toxin (LeTx) neutralization assays demonstrated that the T4-PA immunized animals also elicited robust LeTx neutralization titers (the dilution of serum inducing 50% neutralization [EC_50_] of 4,052 ± 281) (Fig. 3C). The control animals were negative for both types of antibodies (Fig. 3B and C).

The protective efficacy of T4-delivered F1mutV/PA was evaluated by two dual-challenge models that we have recently developed: (i) sequential challenge (Fig. 3D) in which the animals were first exposed to one threat agent and the survivors were then exposed to the second threat agent, and (ii) simultaneous challenge (Fig. 3E) in which the animals were exposed to both threat agents at the same time. For sequential challenge, mice (10 mice per group) immunized as described above (Fig. 3A) were injected intraperitoneally (i.p.) with one 100% lethal dose (LD_100_) of LeTx (1:1 mixture of PA and LF [100 µg each]) on day 42. The immunized group was 80% protected against LeTx challenge, whereas 100% of the negative-control mice died within 2 days of challenge (Fig. 3D). Thirty-three days later, the surviving animals were challenged with 400 50% lethal doses (LD_50_) of Y. pestis CO92 by intranasal (i.n.) administration to develop pneumonic plague. The naive mice were used as negative controls. The T4-F1mutV/PA group showed 100% protection (no death), whereas the naive animals showed 100% death within 4 days after Y. pestis CO92 challenge (Fig. 3D).

For simultaneous dual challenge, mice (*n* = 8) immunized by the same scheme (Fig. 3A) were challenged with both LeTx (1 LD_100,_ i.p. administration) and Y. pestis CO92 (200 LD_50_, i.n. administration) 23 days after the boost (Fig. 3A). As shown in Fig. 3E, all the control mice died within 2 days of challenge, whereas the T4-delivered F1mutV/PA provided 88% protection (one death out of eight mice). Furthermore, the survivors showed clearing of Y. pestis bacteria by 3 days postchallenge (Fig. 3F). The Y. pestis CO92 strain used in the challenge experiment contained a luciferase (*lux*) expression cassette for imaging the bacteria *in vivo* in real time (39). The immunized animals were negative for bioluminescence, whereas the control mice showed bacterial dissemination throughout the body (Fig. 3F); these data were confirmed by colony count determination at the termination of the experiment or as the animals succumbed to infection (data not shown).

### The T4 nanoparticles provide complete protection to rats against both anthrax and plague.

**FIG 4:**
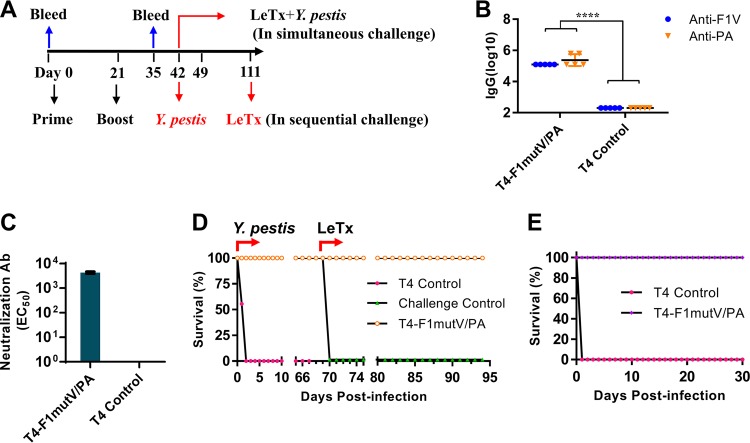
(A) Immunization scheme for rat study. Rats were immunized (i.m.) on days 0 and 21. Sera were collected on days 0 and 35 for antibody analysis. In a sequential challenge model, rats (*n* = 9) were challenged with 400 LD_50_ Y. pestis CO92 (i.n.) on day 42, followed by i.v. injection of 1 LD_100_ LeTx (i.v.) on day 111. In a simultaneous challenge model, rats (in a separate experiment [*n* = 6]) were immunized as shown in panel A and challenged with 1 LD_100_ LeTx (i.v.) and 400 LD_50_ Y. pestis CO92 (i.n.) on day 42. (B and C) Antigen-specific total IgG titers (B) and LeTx neutralizing antibody titers (C). Five serum samples from each group were randomly selected for serum analysis. Error bars represent standard deviations. Values that are significantly different (*P* < 0.0001 by Student’s *t* test) are indicated by a bar labeled with four asterisks. (D) Survival of rats against anthrax LeTx and plague sequential challenge. (E) Survival of rats against simultaneous anthrax LeTx and plague challenges. The animal survival data are representative of four biological replicates.

The rat is a natural host for Y. pestis infection, which occurs through rat fleas. Therefore, rats are one of the most reliable models to assess the protective efficacy of vaccines against plague (40). Likewise, rats are exquisitely sensitive to LeTx (19). To evaluate the immunogenicity and protective efficacy of the T4 bivalent vaccine using this model, Brown Norway rats (*n* = 9) were immunized using the scheme shown in Fig. 4A. The T4-delivered immunogens induced high levels of antigen-specific IgG titers in rats, up to ∼1.25 × 10^5^ and ∼6.25 × 10^5^ of F1mutV-specific and PA-specific IgG, respectively (Fig. 4B). The T4-F1mutV/PA group consistently also generated high levels of LeTx neutralizing antibodies (EC_50_ of 4,285 ± 409) (Fig. 4C). The control animals, as expected, were negative for the antigen-specific total IgG and LeTx neutralizing antibodies (Fig. 4B and C).

The protective efficacy of the T4 bivalent vaccine in rats was also tested by our dual-challenge models (Fig. 4D and E). For sequential challenge, the animals (*n* = 9) were first subjected to i.n. challenge with 400 LD_50_ of Y. pestis CO92. The T4 bivalent vaccine showed 100% protection, whereas all the rats in the control group died within 2 days postchallenge (Fig. 4D). The surviving rats were then challenged with 1 LD_100_ of LeTx (7.5 µg each of PA and LF) by intravenous (i.v.) injection on day 70 after Y. pestis CO92 challenge. All the rats immunized with T4 dual anthrax-plague vaccines survived (Fig. 4D), but rats in the control group (negative control) died within 2 h of the LeTx challenge. This is consistent with previous studies in that rats are highly sensitive to i.v. administration of LeTx, a challenge regime that results in rapid death (19). In a separate experiment, the protective efficacy of the T4 vaccine was further evaluated by the simultaneous dual-challenge model where the rats (*n* = 6) were immunized as described above and challenged with both LeTx (1 LD_100_, i.v.) and Y. pestis CO92 (400 LD_50_, i.n.) at the same time (Fig. 4E). The T4 dual vaccines once again showed 100% protection, whereas the control animals succumbed to either the LeTx challenge or to Y. pestis infection (Fig. 4E).

### The T4 nanoparticle vaccine induces high levels of both T_H_1- and T_H_2-dependent antibody responses.

**FIG 5:**
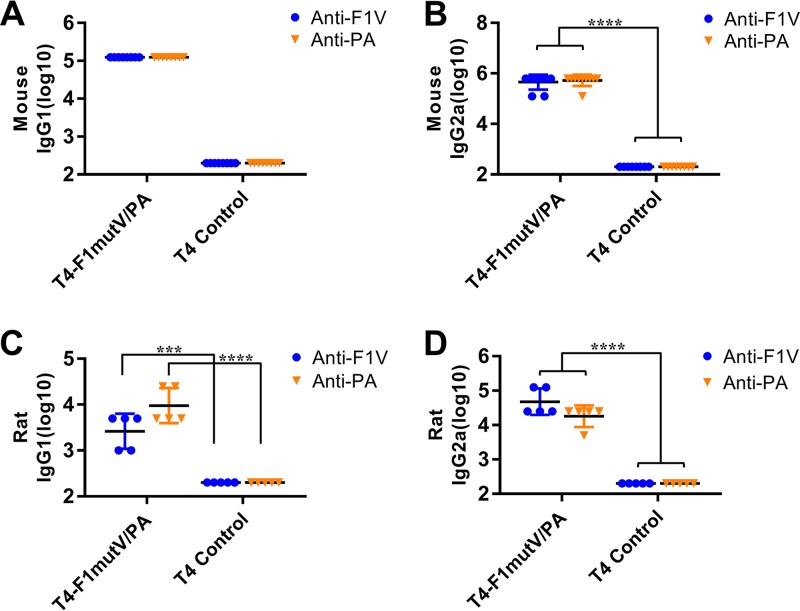
Mouse antigen-specific IgG1 (A) and IgG2a (B) antibody titers and rat antigen-specific IgG1 (C) and IgG2a (D) antibody titers. Animals were immunized (i.m.) according to Fig. 3A and 4A. Sera were collected and analyzed by ELISA. Values that are significantly different by Student’s *t* test are indicated by bars and asterisks as follows: ***, *P* < 0.001; ****, *P* < 0.0001.

Stimulation of both arms of the immune system, humoral (T_H_2) and cellular (T_H_1), is essential for protection against Y. pestis infection (10), and probably beneficial for protection against B. anthracis infection (41). With this in mind, we determined the IgG subclass of the induced antibodies (Fig. 5). In mice, the IgG2a titer represents the T_H_1 response, whereas the IgG1 titer reflects the T_H_2 response. Our data showed that the T4-displayed F1mutV/PA group elicited high levels of both IgG1 and IgG2a antibodies against F1mutV or PA, whereas the control animals were negative for both types of antibodies (Fig. 5A and B). The IgG subclass specificity of the antibodies induced in rats exhibited trends similar to those in mice (Fig. 5C and D). The T4 nanoparticle-displayed F1mutV/PA induced high levels of both IgG1 and IgG2a antibodies against both F1mutV and PA immunogens (Fig. 5C and D). However, a bias toward T_H_1 was even stronger in the case of anti-PA than in the case of anti-F1mutV (Fig. 5C and D). These results, which were consistently observed in two animal models, showed that the T4 nanoparticle-delivered antigens stimulated strong antibody responses derived from both the T_H_1 cellular system and the T_H_2 humoral system (17, 42). In contrast, we and others have previously noted that soluble antigens (with Alhydrogel as an adjuvant) showed a clear bias toward the T_H_2 responses (17, 43). This is also consistent with the clearance of pathogenic Y. pestis *lux* bacteria in the challenge experiments described above. However, more studies are needed to directly examine the T_H_1 and T_H_2 responses and to further explore the mechanisms of T4 nanoparticle vaccine-induced protection.

### The T4 nanoparticles confer complete protection to rabbits against inhalational anthrax.

**FIG 6:**
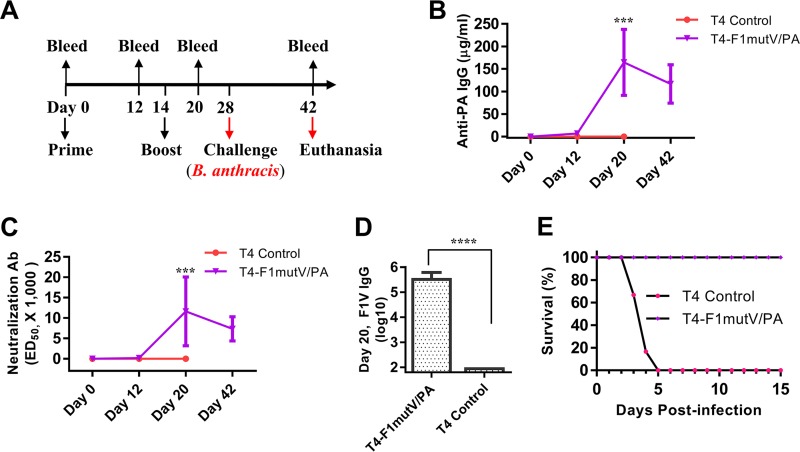
(A) Immunization scheme for rabbit study. Rabbits (10 rabbits in the T4-F1mutV/PA group and 6 rabbits in the control group) were immunized on day 0 and given a boost on day 14. Animals were challenged with 200 LD_50_ of aerosolized B. anthracis Ames spores 2 weeks after the boost. (B) PA-specific total IgG antibody titers. The titers for bleeds on days 0, 12, 20, and 42 are shown. (C) LeTx neutralizing antibody titers. (D) F1V-specific total IgG antibody titers for day 20. Error bars represent standard deviations (SD) of the means. Values that are significantly different by Student’s t test are indicated by bars and asterisks as follows: ***, *P* < 0.001; ****, *P* < 0.0001. (E) Survival of the rabbits challenged with 200 LD_50_ of aerosolized B. anthracis Ames spores. The survival data are from 10 vaccinated rabbits and 6 control rabbits.

The T4 dual anthrax-plague vaccine was further evaluated in a New Zealand White (NZW) rabbit model that is considered to be the best model for inhalation anthrax. The pathology of rabbits challenged with aerosolized Ames spores shows remarkable similarity to that in humans infected with encapsulated toxigenic B. anthracis spores (44, 45). NZW rabbits (10 rabbits per group) were primed on day 0 and boosted on day 14 by the i.m. injection of the T4 bivalent anthrax-plague vaccine formation (Fig. 6A). Sera were collected on the schedule shown in Fig. 6A and subjected to immunological analyses. The data showed that the T4 nanoparticle vaccines induced high levels of anti-PA IgG antibodies as well as LeTx neutralizing antibodies on day 20 (Fig. 6B and C). The rabbits also induced high levels of anti-F1mutV antibodies, up to an endpoint titer of 1.6 × 10^6^ (Fig. 6D). The control group showed no antibodies to either PA or F1mutV (Fig. 6B to D).

Rabbits were challenged 2 weeks after the boost with 200 LD_50_ of aerosolized B. anthracis Ames spores. All the naive control rabbits succumbed to the anthrax disease 2 to 4 days postinfection, while the T4 dual-vaccine-immunized rabbits were 100% protected (Fig. 6E). Blood samples for bacteremia were drawn before the challenge on day 27 and on days 29 to 33 (2 to 6 days postexposure) and 42 (15 days postexposure). Bacteremia was not detected in vaccinated animals, whereas all unvaccinated animals became positive for bacteremia before they succumbed to the disease. To determine the bacterial loads of internal organs, postmortem collection of specimens was performed after scheduled euthanasia of surviving animals on study day 42 (T4-F1mutV/PA group) or after animals died due to the anthrax exposure (control group). All vaccinated animals had cleared the agent from the lungs and did not have any bacteria in the brain, liver, or spleen. In contrast, tissue samples collected from control animals were positive for B. anthracis (except for one rabbit whose liver was negative), indicating systemic anthrax infection.

## DISCUSSION

The deadly anthrax attacks of 2001 using weaponized spores of B. anthracis illustrated the enormous dangers posed by biothreat agents in creating chaos and terror among the public (3, 4). Stockpiling of a biodefense vaccine that can protect people against multiple biothreat agents would provide an effective countermeasure to mitigate such future attacks. Despite intense efforts for nearly 2 decades, no such vaccine is on the horizon for licensure. We describe here a nanoparticle vaccine delivery platform using phage T4 that can be engineered to create multivalent biodefense vaccines against different pathogens. As a proof of principle, a dual anthrax-plague vaccine is formulated by incorporating three different antigens from two Tier 1 select agents into phage T4 nanoparticles; protective antigen from B. anthracis and the capsular protein F1 and the low-calcium-response V antigen from Y. pestis.

The anthrax and plague antigens were assembled *in vitro* on the capsid of phage T4 through fusion to the phage outer capsid protein Soc. High-affinity interactions between Soc and the capsid fixed the antigens at symmetric positions of the capsid lattice. Recent studies showed that repeat structures of the viral capsid could be recognized by Toll-like receptor 2 (TLR-2) as a PAMP and could lead to the induction of an innate immune response (46). Therefore, a simple mixture of anthrax and plague antigen particles, with the displayed antigens resembling the repeat structures or molecular patterns seen on a natural viral pathogen, might then be presented to the host immune system.

Strikingly, the T4 nanoparticle vaccine elicited high titers of antigen-specific antibodies against both anthrax and plague antigens. Neither antigenic interference nor enhancement of antibody responses was evident. Furthermore, the dual vaccine was highly efficacious in protecting animals against lethal challenges with anthrax and/or plague agent. This was observed in three different animal models: BALB/c mice, Brown Norway rats, and New Zealand White rabbits. Indeed, complete protection of vaccinated animals was observed even when the animals were simultaneously challenged with LeTx and the highly virulent Y. pestis CO92 bacteria in the rat model or in an inhalational anthrax model where the rabbits were challenged with the aerosolized Ames spores of B. anthracis. These models represent two of the best models available to assess the efficacy of plague and anthrax vaccines, respectively.

It is significant that the dual anthrax-plague vaccine, unlike the traditional subunit vaccines, elicited robust immune responses against both antigens in the absence of an adjuvant. In fact, addition of adjuvants such as Alhydrogel and/or liposomes to the nanoparticles did not enhance the immune responses (data not shown). We speculate that this might be because the viral nanoparticles, mimicking the PAMPs of a natural pathogen, engage with the TLRs and robustly stimulate the innate and adaptive immune systems of the host and do not need an external adjuvant (46). Consistent with this hypothesis, the T4 dual vaccine elicited both T_H_1- and T_H_2-mediated antibody responses against both antigens. This seems to be a signature characteristic of the phage T4 nanoparticle platform, one that is highly desirable for clearance of pathogenic organisms during natural infection. However, further studies are needed to understand the mechanisms.

In conclusion, our studies highlight some unique properties of the T4 phage nanoparticle that are distinct from the traditional subunit vaccines, which could facilitate formulation of multivalent vaccines against high-risk pathogens. Potentially, additional antigens from other biothreat agents and/or from emerging pathogens could be incorporated using the same principle, and these formulations can be customized to address different threats in different geographical regions of the world. Additional advantages of phage T4 platform include the following: highly stable structure, scalability, cost-effectiveness, safety, and lack of preexisting immunity in humans. Together, these factors could accelerate the streamlining of clinical trials, manufacture, and deployment at a much reduced cost, time, and effort. Phage T4, thus, is a good candidate to develop as a “universal” platform for creating and stockpiling multivalent vaccines as part of our national preparedness against potential future biothreats and emerging infections. With some more refinement, this platform may have the most desirable target product profile for licensure.

## MATERIALS AND METHODS

### Ethics statement.

This study was conducted in accordance with the *Guide for the Care and Use of Laboratory Animals* (47) recommended by the National Institutes of Health. All animal experiments were performed according to the protocols approved by the Institutional Animal Care and Use Committees of the University of Texas Medical Branch, Galveston, TX (Office of Laboratory Animal Welfare assurance number A3314-01), The Catholic University of America, Washington, DC (Office of Laboratory Animal Welfare assurance number A4431-01), and Southern Research Institute, Birmingham, AL (Office of Laboratory Animal Welfare assurance number A3046-01). All of the select agent animal research was conducted in the animal biosafety level 3 (ABSL3) suite, and the principal investigators have registered with the CDC to work with these pathogens.

### Plasmids, bacterial, and phage T4.

The E. coli expression vector pET28b (EMD Biosciences, Darmstadt, Germany) was used for recombinant plasmid construction. Expression plasmid pET-F1mutVSoc was constructed previously (17, 48). The pET-Soc-PA plasmid was constructed by replacing the F1mutV gene of pET-F1mutV-PA (19) with Soc, which was amplified from pET-F1mutVSoc by PCR. The amplified Soc fragment was doubly digested with NheI and HindIII and cloned into pET-F1mutV-PA, linearized with the same enzymes, to replace F1mutV. The resulting pET-Soc-PA contains the Soc gene fused in frame to the N terminus of PA with a short linker (Glu-Ala-Ser-Ala) between the Soc gene and PA. All plasmids were confirmed by sequencing. E. coli strains DH5α was used for cloning. E. coli strain P301 was used to propagate *hoc*^−^ *soc*^−^ phage T4 as described previously (49–51). The E. coli BL21-CodonPlus (DE3)-RIPL cells (Agilent Technologies, Santa Clara, CA) were used for expression of genes encoding target proteins.

### Purification of proteins.

The E. coli BL21-CodonPlus (DE3)-RIPL cells containing either pET-Soc-PA or pET-F1mutVSoc were induced with 1 mM isopropyl-β-d-1-thiogalactopyranoside (IPTG) for 2 to 3 h at 28°C. Recombinant proteins were purified as described previously (17, 48). Briefly, cells were harvested and resuspended in binding buffer (50 mM Tris-HCl [pH 8], 300 mM NaCl, and 20 mM imidazole) containing protease inhibitor cocktail (Roche, USA, Indianapolis, IN). After the cells were lysed at 19,610 lb/in^2^ using a French press (Aminco, Urbana, IL), the soluble fractions of cell lysis containing the His-tagged fusion proteins were isolated by centrifugation. Proteins were purified first by HisTrap column (AKTA-prime; GE Healthcare Bio-Sciences Corp., Piscataway, NJ) followed by size exclusion chromatography on a HiLoad 16/60 Superdex 200 column (AKTA-FPLC; GE Healthcare). The proteins were then quantified and stored at −80°C until use.

### Purification of Hoc^−^ Soc^−^ T4 phage.

Hoc^−^ Soc^−^ T4 phages were purified as described previously (48, 49). Briefly, the propagated T4 phage on E. coli P301 was collected by centrifugation for 45 min at 25,000 × *g*. The pellet containing T4 phages was resuspended in 40 ml Pi-Mg buffer (26 mM Na_2_HPO_4_, 68 mM NaCl, 22 mM KH_2_PO_4_, 1 mM MgSO_4_ [pH 7.5]) containing 10 µg/ml DNase I and chloroform (0.4 ml) and incubated at 37°C for 30 min. The lysate was subjected to low-speed centrifugation (6,000 × *g* for 10 min) and high-speed centrifugation (35,000 × *g* for 45 min), and the final phage pellet was resuspended in 200 µl of Tris-Mg buffer (10 mM Tris-HCl [pH 7.5], 50 mM NaCl, and 5 mM MgCl_2_) and purified by CsCl density gradient centrifugation.

### *In vitro* binding of antigens to phage T4.

*In vitro* binding of Soc fusion proteins to Hoc^−^ Soc^−^ T4 phage was conducted as previously described (17, 48). The same batch of purified phages was used for all the animal immunization experiments (below). The proteins and phages used were highly pure, after going through multiple rounds of purification steps as described above. As previously reported (17), the endotoxin levels in three different batches of purified proteins ranged from 0.05 to 0.8 endotoxin unit (EU) per ml, well below the maximum recommended endotoxin levels, 10 and 20 EU/ml, in gene vectors and subunit vaccines, respectively (52). Moreover, the T4 nanoparticle vaccines used for immunizations had undergone additional purification during the *in vitro* binding reaction where the displayed particles were washed twice with excess phosphate-buffered saline (PBS) to remove the unbound antigen and any other minor contaminants. Briefly, F1mutV-Soc or Soc-PA proteins were incubated with purified Hoc^−^ Soc^−^ phage at 4°C for 45 min. The phage particles containing the bound proteins were centrifuged at 34,000 × *g* for 45 min. After two washes with excess PBS buffer (pH 7.4) to remove unbound proteins, the final phage pellets containing the bound antigens were resuspended in PBS buffer (pH 7.4) and analyzed by SDS-PAGE using Novex 4-20% Tris-Glycine Mini Gels (Thermo Fisher Scientific, Waltham, MA).

The copy number of displayed antigen per capsid was calculated by quantifying the density of Coomassie blue-stained Soc fusion bands and the internal control band, T4 gp23*, using the Bio-Rad ChemiDoc MP imaging system. Each lane was individually quantified to minimize any staining differences. Since the copy number of the major capsid protein gp23* (molecular weight [MW] of 49 kDa) was established to be 930 per capsid and that of the tail sheath protein gp18 (MW of 72 kDa) was established to be 138 per phage and since we used 2 × 10^10^ particles in 200-µl reaction mixture, we could compute the copy number of bound antigen per capsid. However, keeping in mind differences in the staining densities of proteins and quantification of pixel densities, we estimated experimental variation to be within twofold. Further, the data shown are consistent with the copy numbers determined in previous studies using phage T4 Soc-PA (35) and samples prepared for many immunization experiments performed as part of this study. For Fig. 2, the copy number is shown on the *y* axis at each of the ratios used. The concentration of the free antigen (*x* axis) was determined by subtracting the capsid-bound antigen from the total antigen added to the reaction mixture. Saturation binding curves (Fig. 2B and D) were then generated from these data. The *B*_max_ and BC_50_ values were determined by nonlinear regression analysis using GraphPad Prism-7 software (San Diego, CA). BC_50_, a measure of binding affinity, is defined as the molar concentration of Soc fusion protein (ligand) at which half the available capsid binding sites are occupied by the ligand. *B*_max_ is defined as the maximum number of binding sites occupied by the displayed Soc fusion protein per capsid particle as determined from the saturation binding curve.

### Mouse immunizations and challenges.

Six- to eight-week-old female BALB/c mice (17 to 20 g) purchased from The Jackson Laboratory (Bar Harbor, ME) were randomly assigned to groups and allowed to acclimate for 7 days. Antigens were displayed on T4 as described above. A total of 50 µg antigen (25 µg of each F1mutV and PA) was injected on days 0 and 21 via the i.m. route. Control mice received the same amount of T4 but without any antigen. Blood samples were collected from each animal by the retro-orbital route on day 0 (prebleeds) and day 35 for immunological analyses. Mice were sequentially or simultaneously challenged with LeTx and Y. pestis CO92 as described previously (19). Briefly, in sequential challenge, mice were i.p. challenged first with 1 LD_100_ of LeTx, followed by i.n. challenge with 400 LD_50_ (1 LD_50_ = 100 CFU in BALB/c mice) of Y. pestis CO92 33 days after LeTx challenge. In a separate simultaneous challenge experiment, mice were immunized as described above and i.p. challenged with 1 LD_100_ of LeTx, followed by i.n. challenge with 200 LD_50_ Y. pestis CO92 on the same day. All animals were anesthetized by inhalation of 2% to 4% isoflurane (to effect) before challenge. Animals were monitored twice daily for mortality and other clinical symptoms.

### Rat immunizations and challenges.

Female Brown Norway rats (50 to 75 g) were purchased from Charles River Laboratories (New Jersey) and randomly grouped. After 7 days acclimation, rats were immunized by intramuscular (i.m.) route with T4-displayed antigens prepared as described above. Twenty-five micrograms of each antigen was used for immunizations as indicated in the figures. The animals were bled on day 35 by the saphenous vein, and sera were obtained for immunological analyses. Rats were sequentially challenged on day 42 with ∼400 LD_50_ Y. pestis CO92 (i.n.) followed by 1 LD_100_ LeTx (7.5 µg of each of the toxin components [LF and PA]) challenge (i.v.). In a separate simultaneous challenge experiment, rats (six rats per group) were immunized as described above and challenged simultaneously with 1 LD_100_ of LeTx and 400 LD_50_ Y. pestis CO92 3 weeks after boost. All rats were anesthetized by inhalation of 2% to 4% isoflurane (to effect) before challenge. Rats were monitored twice a day for morbidity and mortality.

### Rabbit immunization and challenge.

The rabbit study was conducted by the Southern Research Institute (study number 13538.01.15; Birmingham, AL). A total of 16 New Zealand White rabbits were randomly divided into two groups. Group 1 was vaccinated with T4-displayed antigen (50 µg) (10 rabbits, equal numbers of males and females), while group 2 received the same amount of T4 but without any antigen (6 rabbits, equal numbers of males and females). Rabbits were immunized on days 0 and 14. Sera were collected on days 0 (preimmune), 12, 20, and 42 for immunological analyses. For the challenge experiment, the animals were loaded in the head-out plethysmograph, and a custom designed nose-only inhalation challenge mask was placed over the snout of each rabbit so that the mouth and nares were covered. At the start of the challenge period, the nebulizer and liquid impinger were actuated. Animals received the aerosol challenge until a cumulative inhaled volume of 20 liters had been reached. The inhalation exposure time was 11.7 to 22.8 min. Animals were challenged with 200 LD_50_ of aerosolized B. anthracis Ames spores on day 28 and monitored for morbidity and mortality until day 42. The remaining animals were then euthanized by an intravenous administration of a barbiturate overdose, and tissues (brain, liver, lung, and spleen) were collected for B. anthracis detection on the same day. Blood samples (approximately 0.2 ml) were also collected on days 27, 29 to 33, and 42 for microbiological analysis (bacteremia).

### Determination of IgG and IgG subtype antibodies.

Antibody titers were determined by ELISA as described previously (17, 42). Briefly, each well of a 96-well plate was coated with 100 ng of F1mutV or PA diluted in coating buffer (0.05 M sodium carbonate-sodium bicarbonate [pH 9.6]) overnight at 4°C. The plates were then blocked with 3% bovine serum albumin (BSA) in PBS (pH 7.4) for 1 h at 37°C. After 1 h of incubation at 37°C with serially diluted serum samples, plates were washed with PBS-T (PBS with 0.1% Tween 20 [pH 7.4]). For total IgG, horseradish peroxidase (HRP)-conjugated goat anti-mouse IgG (KPL, Gaithersburg, MD), rabbit anti-rat IgG (Invitrogen), or goat anti-rabbit IgG (KPL, Gaithersburg, MD) was used as the secondary antibody. For mouse or rat IgG subtypes, HRP-conjugated goat anti-mouse or HRP-conjugated mouse anti-rat IgG1 or IgG2a secondary antibodies (Abcam, Cambridge, MA) were used. Samples were initially diluted 1:200; serial fivefold dilutions were performed as necessary to ensure that values reached the endpoint. For rabbit anti-PA IgG titers, plates were coated with PA, and affinity-purified rabbit anti-PA polyclonal antibody was used to generate a standard curve, from which the sample anti-PA IgG concentrations (in nanograms per milliliter) were determined. 3,3′,5,5′-Tetramethylbenzidine (TMB) microwell peroxidase substrate kit (KPL, Gaithersburg, MD) was used for staining.

### Anthrax LeTx neutralization assay.

Anthrax lethal-toxin-neutralizing assay was performed as described previously (53). Briefly, PA and LF were diluted to the final concentration of 200 ng/ml with Dulbecco’s modified Eagle’s medium (DMEM). Serially diluted serum samples were added to the toxin mixture, incubated for 1 h at 37°C, and then transferred to RAW264.7 macrophage cells grown to confluence in 96-well plates and incubated for 5 h. The viability of cells was assessed by incubation with MTT [3-(4,5-dimethylthiazo-2-yl)-2,5-diphenyltetrazolium bromide] (Sigma, St. Louis, MO) at a final concentration of 0.5 mg/ml for 30 min. The medium was aspirated, the insoluble pigment (formazan) produced by living cells was dissolved by adding a solution containing 0.5% SDS, 25 mM HCl, and 90% isopropanol, and the optical density (570 nm) was measured to assess viability. The effective serum concentration inducing 50% neutralization (EC_50_) was calculated with Graphpad Prism-7 software (San Diego, CA).

### Live-animal imaging.

*In vivo* imaging was performed as described previously (39). Briefly, 3 days after challenge with Y. pestis CO92-luciferase strain, the animals were imaged by using an IVIS 200 bioluminescence and fluorescence whole-body imaging workstation (Caliper Corp., Alameda, CA) in the ABSL-3 at the University of Texas Medical Branch (UTMB) facility after lightly anesthetizing the animals with isofluorane.

### Statistical analyses.

Results are expressed as means ± standard deviations (SD). Statistical comparisons between groups were evaluated by Student’s *t* test. The animal mortality data were analyzed by the Kaplan-Meier survival estimate. A *P* value of <0.05 was considered statistically significant.
