# Supplementary figures and images for: A Bacteriophage T4 Nanoparticle-Based Dual Vaccine against Anthrax and Plague
Source: mBio. 2018 Oct 16;9(5):e01926-18. doi: 10.1128/mBio.01926-18 (PMC6191538; doi:10.1128/mBio.01926-18)

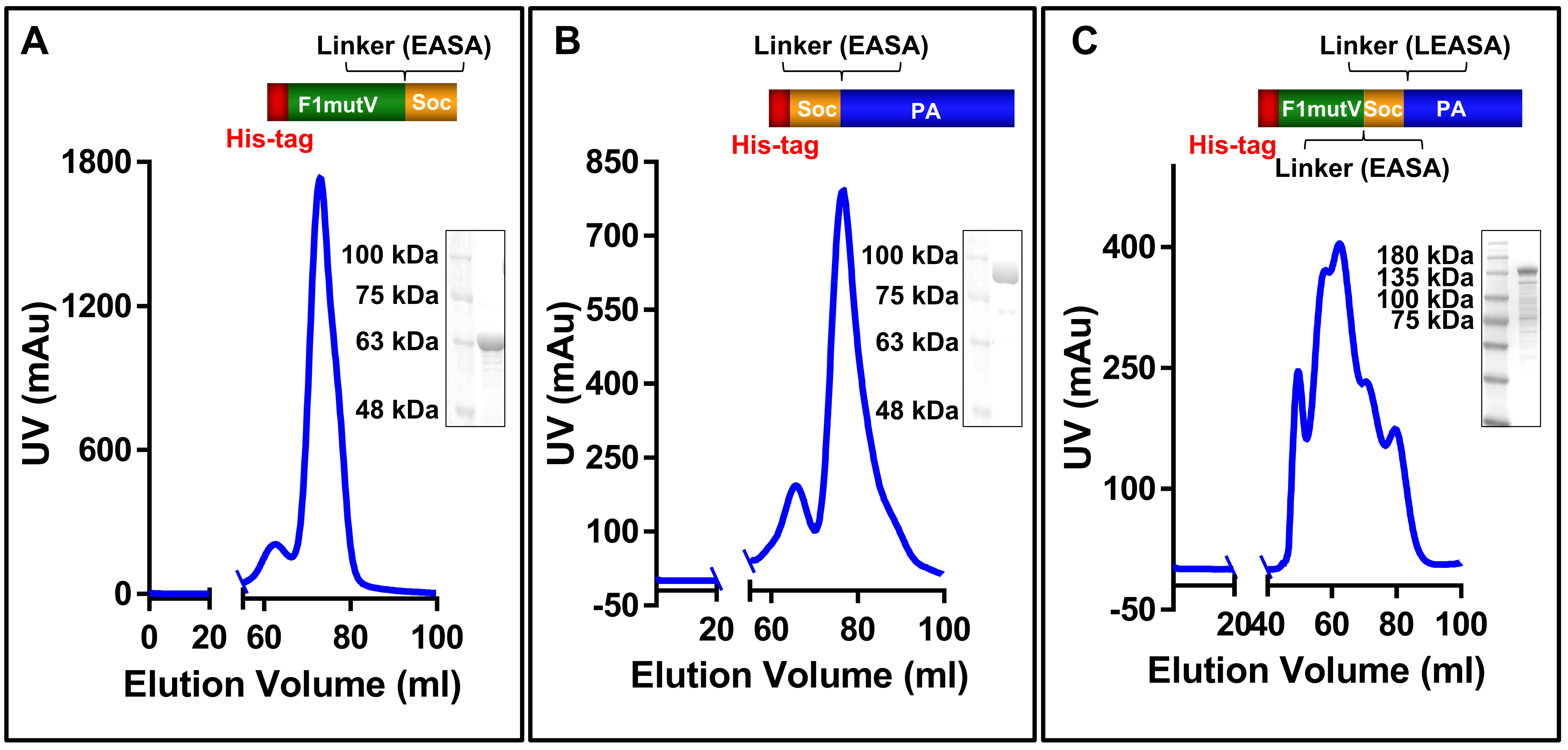

Supplement: FIG S1 [file mbo005184105sf1.tif]
